# Optimal omegas – barriers and novel methods to narrow omega-3 gaps. A narrative review

**DOI:** 10.3389/fnut.2024.1325099

**Published:** 2024-02-02

**Authors:** Emma J. Derbyshire, Catherine S. Birch, Graham A. Bonwick, Ashley English, Phil Metcalfe, Weili Li

**Affiliations:** ^1^Nutritional Insight Limited, London, United Kingdom; ^2^AgriFood X Limited, York, United Kingdom; ^3^HTC Group Limited, Cheshire, United Kingdom; ^4^Efficiency Technologies Limited, Milton Keynes, England, United Kingdom; ^5^University of Chester, Chester, United Kingdom

**Keywords:** bioavailability, delivery methods, docosahexaenoic acid, eicosapentaenoic acid, food fortification, long-chain polyunsaturated fatty acids, microalgae, microencapsulation

## Abstract

Dietary intakes of omega-3 long chain polyunsaturated fatty acids (O3LC-PUFAs) such as eicosapentaenoic and docosahexaenoic acid are central to development and health across the life course. O3LC-PUFAs have been linked to neurological development, maternal and child health and the etiology of certain non-communicable diseases including age-related cognitive decline, cardiovascular disease, and diabetes. However, dietary inadequacies exist in the United Kingdom and on a wider global scale. One predominant dietary source of O3LC-PUFAs is fish and fish oils. However, growing concerns about overfishing, oceanic contaminants such as dioxins and microplastics and the trend towards plant-based diets appear to be acting as cumulative barriers to O3LC-PUFAs from these food sources. Microalgae are an alternative provider of O3LC-PUFA-rich oils. The delivery of these into food systems is gaining interest. The present narrative review aims to discuss the present barriers to obtaining suitable levels of O3LC-PUFAs for health and wellbeing. It then discusses potential ways forward focusing on innovative delivery methods to utilize O3LC-PUFA-rich oils including the use of fortification strategies, bioengineered plants, microencapsulation, and microalgae.

## Introduction

**Figure 1:**
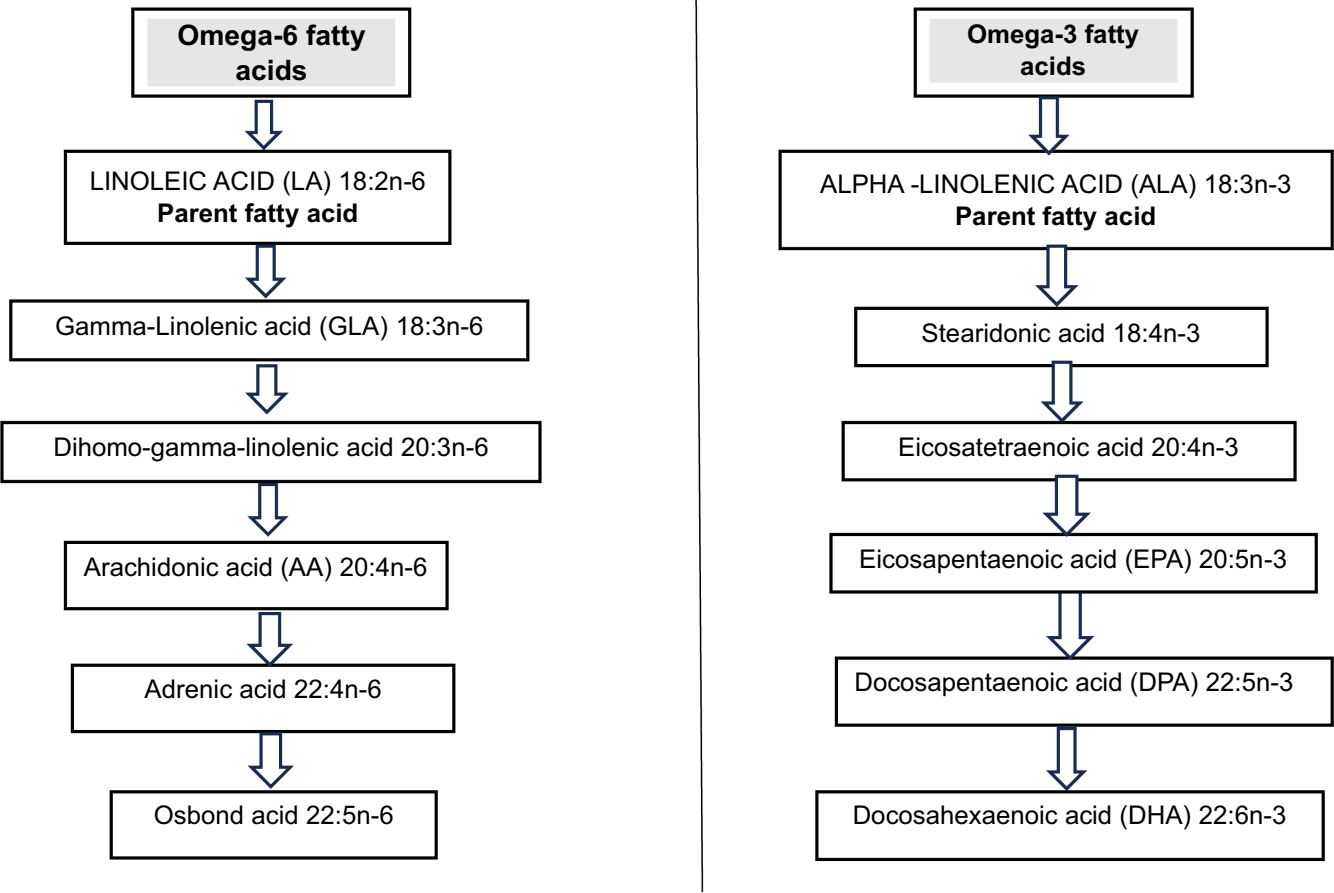
Omega-6 and omega-3 metabolic pathways. Source: Adapted from Miles and Calder (2) and Bird et al. (3).

Omega-3 long chain polyunsaturated fatty acids (O3LC-PUFAs) include α-linolenic acid (ALA; 18:3), stearidonic acid (SDA; 18:4), eicosatetraenoic acid (20:4), eicosapentaenoic acid (EPA; 20:5), docosapentaenoic acid (DPA; 22:5), and docosahexaenoic acid (DHA; 22:6) (1) (Figure 1). One means of assessing the conversion rate of ALA to the longer-chain products EPA, DPA and DHA is to determine the net rise in blood circulating levels of these fatty acids after increasing ALA intakes in controlled human trials. Isotope studies by Burdge and Calder (4) have demonstrated that ALA seems to be a limited source of O3LC-PUFAs, particularly for males although estrogen may improve the conversion of ALA to O3LC-PUFAs in women which could be a regulatory mechanism to meet fetal and neonate DHA needs. Other research using isotope labeled fatty acids in young women found that estimated ALA inter-conversion was 21% for EPA and just 6% for DPA and 9% for DHA (5).

Regarding omega-6 long chain polyunsaturated fatty acids linoleic acid (the parent omega-6 fatty acid) yields gamma-linolenic acid, dihomo-gamma-linolenic acid, arachidonic acid, adrenic acid and then osbond acid (2). It was George and Mildred Burr in 1929 who first deciphered the “essentiality” of these fatty acids when rodent models were fed a fat-free diet (6). They identified that fatty acids were essential nutrients and found that the omega-6 fatty acid linoleic acid prevented disease thus was an ‘essential’ fatty acid (7). They also found that linolenic acid (the omega-3 analog of linoleic acid) was also an essential fatty acid (7). Linoleic acid and ALA acid cannot be produced endogenously by humans (mammals) thus are often termed ‘essential’ fatty acids although it should be considered that these fatty acids can be manufactured by plants and are found in plants tissues such as seeds, seed oils and nuts (2).

Physiologically, the omega-3 fatty acids are central building blocks of cell membranes (8). Around 50–60% of the brain weight consists of lipids and of this about 35% is derived from O3LC-PUFAs, with DHA accounting for over 40% of omega-3 fatty acids in the gray matter and neuronal tissue (9, 10). The function and structure of the human brain is dependent on an adequate and constant supply of EPA and DHA. O3LC-PUFAs have been found to play key roles in cell membrane fluidity, human growth and development, vision, reduced breast and colorectal cancer risk, lower metabolic syndrome risk and are known to exert cardio-protective actions (11–13). Meta-analytical evidence also shows that both O3LC-PUFAs and fish have been linked to reductions in the development of mild cognitive decline and Alzheimer’s disease (14). DPA is also part of healthy nutrition with infants obtaining this from human milk and oily fish, fish oil supplements and grass-fed beef being some of the most predominant sources of DPA in the general population (15). Due to its similar chemical structure to EPA and DHA, DPA has also been linked to improvements in aspects of human health including neural health, lipid metabolism, reduced platelet aggregation and the attenuation of chronic inflammation (15). Evidence is accruing to suggest that EPA, DPA and DHA have both independent and shared effects, particularly in relation to neuroprotective effects (16). DHA remains to be the most quantitatively important O3LC-PUFAs in the human brain but DPA and EPA may act as useful anti-inflammatory mediators (16).

In 2022 the International Society for the Study of Fatty Acids and Lipids (ISSFAL) concluded that O3LC-PUFAs EPA and DHA play a central role in determining gestational length and adequate intakes, consistent with national guidelines, should be achieved in early pregnancy to lower preterm delivery risk (17). A Cochrane report compiled of 70 randomized controlled trials (RCTs) also concluded that preterm births (< 37 weeks) and early preterm births (< 34 weeks) were reduced in women receiving O3LC-PUFAs compared with no omega-3 (18). Possible reduced risks of neonatal care admission, perinatal death, low birthweight, and slight elevated risk of large for gestational age infants were also found for those receiving O3LC-PUFAs (18).

In the last 5 years several Cochrane reports have been published looking at the role of O3LC-PUFAs in relation to health (Table 1). Evidence appears to be stronger for certain health outcomes such as coronary heart disease and depressive symptomology than for others (20, 21). Overall, the health benefits of O3LC-PUFAs are becoming increasingly recognized. Despite this, O3LC-PUFA intakes in many regions and populations globally remain to be inadequate. The present narrative review describes this, discusses barriers to obtaining suitable levels of O3LC-PUFAs and reviews potential ways forward; focusing on innovative delivery methods to use O3LC-PUFA-rich oils including the use of fortification strategies, bioengineered plants, microencapsulation, and microalgae.

**Table 1:** Summary of evidence from Cochrane reports focusing on O3LC-PUFAs and aspects of health (last 5 years).

Reference	Aspect of Health/Area of Focus	Number of RCTs/studies reviewed	Sample size evaluated (*n*)	Main findings
Gillies et al. (19)	Attention deficit hyperactivity disorder	37	2,374	There was low-certainty evidence that children and adolescents receiving O3LC-PUFA were more likely to improve compared to those receiving placebo
Appleton et al. (20)	Major depressive disorder (adult focus)	35	1924	Analysis suggests a small-to-modest, non-clinically beneficial effect of O3LC-PUFA on depressive symptomology vs. placebo. The certainty of the evidence was low to very low
Abdelhamid et al. (21)	Primary and secondary prevention of cardiovascular disease	86	162,796	Moderate- and low-certainty evidence. Increasing O3LC-PUFAs slightly reduces risk of coronary heart disease mortality and events and reduces serum triglycerides (evidence from supplement trials). Increasing ALA slightly reduces risk of cardiovascular events and arrhythmia
Watson & Stackhouse (22)	Cystic fibrosis	23	106	Regular O3LC-PUFA supplements could provide some limited benefits for people with cystic fibrosis with few adverse effects. The evidence quality was very low
Dushianthan et al. (23)	Acute respiratory distress syndrome	10	1,015	Findings of this review were limited by lack of standardization among the included studies with regard to types of nutritional supplements given, methods and reporting of outcome measures. The evidence quality was low to very low.
Downie et al. (24)	Dry eye diseases	34	4,314	There is a possible role for O3LC-PUFA supplementation in managing dry eye disease, although the evidence is inconsistent and uncertain

## Human recommendations

Several organizations and publications have established recommendations for O3LC-PUFAs. This advice is summarized in Table 2. The European Food Safety Authority (EFSA) in 2012 published guidance and recommendations in relation to Tolerable Upper Intake Levels for EPA, DHA and DPA (the maximum daily intake unlikely to cause adverse health effects) (25).

**Table 2:** Summary of O3LC-PUFAs recommendations.

Organization	Recommendation
EFSA (25)	Dietary recommendations for EPA and DHA based on cardiovascular risk considerations for European adults are between 250 and 500 mg/day
EFSA (25)	Supplemental intakes of DHA alone up to about 1 g/day do not raise safety concerns for the general population
EFSA (2012) (25)	Supplemental intakes of EPA alone up to 1.8 g/day, do not raise safety concerns for adults
EFSA (25)	Supplemental intakes of EPA and DHA combined at doses up to 5 g/day do not raise safety concerns for adults
Burns-Whitmore et al. (26)	Vegans should have separate AIs for LA and ALA than omnivores and utilize recommendations of between 2.2–4.4 g/g of ALA d (or 1.1 g/day/1000 Kcals)
ISFAAL (17)	Supplementation with a total of about 1,000 mg of DHA plus EPA is effective at reducing risk of early birth, preferably with supplementation commencing before 20 weeks’ gestation

EFSA advised that EPA and DHA recommendations based on cardiovascular risk considerations for European adults are between 250 and 500 mg/day and that supplemental intakes of EPA and DHA combined at doses up to 5 g/day do not raise safety concerns for adults (25). More recently, ISSFAL (17) issued a statement reporting that there is now strong evidence that a proportion of preterm births could be prevented by increasing maternal dietary intakes of O3LC-PUFAs during pregnancy, advising that supplementation with 1,000 mg of DHA plus EPA could lower risk of early birth, ideally with supplementation commencing before 20 weeks’ gestation.

Regarding oily fish consumption, in the United Kingdom it is advised that a healthy and balanced diet should provide 2 weekly portions of fish, of which one should be oily (27). A portion is defined as about 140 g (4.9 oz) (27). Girls, those planning a pregnancy, or who are pregnant/breastfeeding should not eat more than 2 portions of oily fish a week (27). Young people aged 18–25 years (83%) appear to recognize to some extent that EPA and DHA are linked to brain and heart health (28) but they may be less familiar with the specifics, such as intake recommendations for omega-3 fatty acids and oily fish. As seen in studies in the next section, habitual omega-3 and oily fish intakes are generally lower than recommended.

## Intakes of O3LC-PUFAs

Some recent publications have reported on habitual intakes of DHA/EPA and/or blood status. A systematic review of studies (12 reported on total n-3 intakes and eight on EPA and DHA intake) undertaken in Europe, North America and South/East Asia concluded that EPA and DHA intakes were lower amongst those following plant-based diets, defined as vegetarians and vegans in the analysis (29). Within the analysis 13 studies reported on EPA and/or DHA status, with most identifying lower EPA and DHA status in vegetarians and vegans compared to meat-consumers. Vegans also tended to have lower EPA and DHA status than vegetarians (29).

In the United Kingdom a secondary analysis of the National Diet and Nutrition Survey (NDNS), the country’s largest cross-sectional dietary analysis, showed that only a quarter of the UK population were oily fish consumers – a predominant source of omega-3 fatty acids (30). Amongst those consuming oily fish only 7.3% of children, 12.8% of teenagers, and 15.6% of young adults aged 20–29 years fell in line and met oily fish recommendations (30). A further analysis of survey data within this publication (data from 10 publications) found that EPA and DHA intakes were lower than guidelines, with children, teenagers, females, and pregnant women presenting some of the largest dietary gaps (30). The PEAR (31) study conducted with 598 women before/during pregnancy found that women who ate fish prior to pregnancy reduced their intakes of both oily and white fish during pregnancy, with some avoiding it altogether. Overall intakes of fish were lower than intakes advised during pregnancy (36% compliance for consumers pre-pregnancy) (31).

In the United States (US) recent research measured EPA and DHA blood concentrations across pregnancy (32). Women self-reported their dietary intakes at enrolment (13–16 weeks into pregnancy) and at 36 weeks into gestation (32). It was found that polyunsaturated fatty acid consumption decreased from early to late pregnancy which was attributed to a decline in the nutritional quality of diets as pregnancy progressed (32). This is interesting given that sex hormones and physiological changes associated with pregnancy can increase liver enzymes involved in DHA synthesis (33). Levels of EPA and DHA are also ‘more in demand’ in the later stages of pregnancy and exposure to lower *in utero* ω-3 PUFA concentrations has been associated with reduced brain volume in childhood (34). These findings are therefore concerning and imply that EPA and DHA intakes were insufficient which could have extended ramification. Data from an earlier US National Health and Nutrition Examination Survey (NHANES) similarly found that 68% adults and 95% children had long-chain omega-3 levels below recommended thresholds (35).

## Omega barriers

### Endogenous synthesis & bioavailability

It has already been explained how linoleic acid and alpha-linolenic acid are not synthesized by mammals hence termed “essential fatty acids” but can be manufactured by plants (2). Although these fatty acids are not produced endogenously by humans they can be metabolize to other fatty acids (Figure 1) (2).

Some sex differences have been observed with regard to EPA/DHA status and incorporation into blood plasma, cells, and tissues, with this appearing to be slightly higher for females but not to a level that warrants movements towards sex-specific omega-3 dietary recommendations (36). It is thought that sex hormones such as estrogen and progesterone could, in part, be one mechanism behind this, with women tending to have heightened increases in EPA status after ALA supplementation when compared to men (33).

Other factors such as the lipid form in which the omega-3 fatty acid acts can also impact on bioavailability, nutrient delivery, and health. Research by Ghasemifard et al. (37) concluded that the bioavailability of different O3LC-PUFA forms appeared to be highest in the free fatty acid form and lowest in the ethyl ester form with no conclusions drawn from human data in relation to triacylglycerols or phospholipids. Other factors such as matrix effects (capsule ingestion with simultaneous intake of food, food fat content) or galenic form (i.e., emulsification, microencapsulation) can also influence the bioavailability of O3LC-PUFA (38).

### Personal choice

There are an array of reasons why populations may not be obtaining suitable daily intakes of oily fish and omega-3 fatty acids. Amongst pregnant mothers, risk aversion, availability, cost, smell/taste, family preferences and confusions over the number of advised weekly portions and finer details of public health guidance have all been found to act as barriers to fish consumption (31). Research undertaken with older Australian adults (*n* = 854, 51 years+) found that cost was the most frequently reported barrier to fresh finfish consumption (reported by 37%) (39). When sampling fish oils unpleasant after tastes have also been reported (40). In trials with oxidized fish oils, even when added at low levels to yogurt, they could be identified by consumers who reported negative impacts on acceptability. However, they remained interested in consuming such products if there were known health benefits (41). Alongside this, unfavorable aftertastes and challenges swallowing supplements may act as further barriers to suitable omega-3 intakes (42).

Dietary preferences such as vegetarianism or veganism can also impact on O3LC-PUFA intakes. A cross-sectional study of meat-eaters, vegetarians and vegans in the United Kingdom has shown that O3LC-PUFA intakes in non-fish-consumers were 57–80% that of fish-eaters (43). Similarly, the exclusion of oily fish has been has been associated with lower EPA and DHA status in vegetarian women, including the breast milk and infants of vegetarian mothers (44). The bioconversion of the omega-3 parent fatty acid ALA into EPA and then DHA is also inefficient therefore limiting the potential effects of ALA supplementation from vegetarian sources such as flaxseed oil (45, 46).

### Fish source

The EPA and DHA profiles of oily fish can depend on where these are sourced from. For example, in farmed salmon the profile of saturated, monounsaturated and polyunsaturated fatty acids has been found to be 15.0, 55.4, and 29.6%, respectively and 26.3, 47.4, and 26.3% in wild salmon (47). A study feeding fish reared in sea cages with an average start weight of 275 g four diets containing different amounts of EPA and DHA found that fish fed on the higher 3.5% EPA and DHA diet had improved growth, filet visual color and quality compared with those on the three lower EPA and DHA diets (48). A study analyzing the DPA and EPA content of 39 Indian food dishes containing fish recognized that fish biodiversity can affect their nutritional (EPA/DHA) profile and found that *Tenualosa ilisha*, *Sardinella longiceps*, *Nemipterus japonicus*, and *Anabas testudineus* were some of the most abundant sources of DHA and EPA (49).

### Sustainability

Over the past few decades fish has been regarded as key human health asset, contributing to more than 20% of animal protein intake for approximately 3 billion people, with this set to rise with growing populations (50). The promotion of oily fish consumption for health may be viewed as a somewhat ‘antagonistic policy’ where nutrition policies, i.e., eat more oily fish for health has subsequent environmental ramifications such as declining fish stocks. For example, over-fishing is responsible for the decline in freshwater fish and natural marine populations, so much so that since 1995 fishing has been banned for 2–3 months in specific periods of the year in China, to help replenish fish populations (51). Along the African coast near to the equator the dual effects of industrial fisheries and climate change have been hampering fish stocks and ecosystems (52). Concerningly, marine ecologists have projected that fish stocks could collapse by 2050 and emphasize that fishing restrictions and ‘no-take’ zones are of central importance to restore marine ecosystem health (50).

### Contaminants

Oily seafood alongside providing O3LC-PUFAs, and micronutrients may also contain polychlorinated dibenzo-p-dioxins/polychlorinated dibenzofurans (PCDD/Fs), dioxin-like-polychlorinated biphenyls (dl-PCBs) and dioxin-like compounds (DLCs) which are known to pose health risks (53). For some populations, such as pregnant women balancing the benefits of fish consumption against mercury intake can be challenging (54). Rather concerningly, pollutants (erythrocyte mercury and urinary arsenobetaine) have been found to be an affective marker of seafood intake amongst pregnant women, more so than O3LC-PUFAs (55). Projected models have shown that methylmercury levels, a renowned neurotoxicant increased by up to 23% between the 1970s and 2000s, due to dietary shifts initiated by overfishing and are estimated to rise by 56% in Atlantic bluefin tuna due to temperatures rises which can increase organic-matter run off into ocean’s (56).

### Microplastics

The amount of literature now available related to microplastic in fish has been accruing. It is now known that microplastic contamination can occur in nearly all types of aquatic habitats around the globe, with fish being very vulnerable to the ingestion of microplastics (57). Over 690 marine species appear to have been affected by plastic debris, with this number set to rise (58). It has been found that fish unintentionally ingest microplastics, sucking these passively in microfibers whilst breathing, with these being found in the gills and gastrointestinal tracts of fish and having an increase presence in food (59).

Recent studies by Ragusa et al. (60, 61) have identified microplastics in human placental tissue including chorioamniotic membranes and within the syncytiotrophoblast, however, it is yet to be determined whether these microplastics derived from fish intake among pregnant females. These microplastics found in human placentas could contribute to the initiation of pathological processes, such as oxidative stress, apoptosis and inflammatory processes. In various environments globally microplastics (size around<5 mm) have been found to be present (59). From a human health stance certain microplastics such as bisphenol A (BPA), nonylphenol (NP), octylphenol (OP) and polybrominated diphenyl ethers (PBDE) may be potentially toxic to humans (62).

## Potential ways forward

### Fortification

Where there are nutritional shortfalls food fortification can be a cost-effective strategy that can also convey economic, health and social benefits (63). Fortification *per se* has been defined by the 1987 Codex General Principles for the Addition of Essential Nutrients to Foods as “*the addition of one or more essential nutrients to a food whether or not it is normally contained in the food, for the purpose of preventing or correcting a demonstrated deficiency of one or more nutrients in the population or specific population groups*” (64). There are two predominant forms of mainstream fortification – targeted fortification for subpopulations, e.g., infant cereals and market-driven mass fortification with iodine fortification of salt being one example of this (65). Providing that specific food vehicles are used and explicit consumer needs addressed voluntary fortification can play a central role in contributing to nutritional requirements, particularly where gaps are evident (65). Biofortification is another form of fortification that stems back to agronomic practices and has been defined by the World Health Organization as “*the process by which the nutritional quality of food crops is improved through agronomic practices, conventional plant breeding, or modern biotechnology”* (66). These methods therefore aims to improve nutrient levels using agronomic practices rather than manual measures (66).

When it comes to O3LC-PUFAs, food fortification appears to be a growing market sector. In the United States when the Food and Drug Administration first authorized O3LC-PUFA use in supplements the market for such ingredients expanded by 24%, demonstrating their potential for popularity (67). Several past studies have fortified a range of foods (milk, margarines, sausages, luncheon meat, French onion dips, yoghurts) with fish oils (68–72). The majority of these methods demonstrate beneficial changes in omega-3 intakes, EPA and DHA pools and the ω-3 index of erythrocyte membranes (68–70, 72) although a degree of masking using flavors may be needed to override fishy tastes and flavors (68). It is worth mentioning that some studies use the terminology “enriched” rather than fortified.

## Bioengineered plants

Alongside marine and plant oils genetic modifications of plants is being explored as a novel ways to produce and supply EPA and DHA (73). Scientists are now able to modify endogenous genes involved in the biosynthesis pathways, enabling the modifications of edible plant oils to upregulate and increase the content of desired components (such as omega-3 fatty acids) or reduce the content of undesirable components (74).

In particular, the transfer of certain genes into plants (such as oilseeds) from microalgae is being viewed as a promising and potentially effective way to yield these fatty acids (73). Omega-3 fish oil crops have also been produced although a range of challenges have also been presented which include underlying metabolic engineering, crop performance, intellectual property, regulatory and consumer acceptance difficulties (75).

## Microencapsulation

Microencapsulation is a novel technology that has been developed with the intention of protecting sensitive compounds from environmental elements which also includes protecting compounds from digestive enzymes for enhanced delivery to the gut with minimal degradation (76). This method has been well used in pharmaceutical sectors and there is now rising interest in its application in food systems (76). Microencapsulation may have many potential uses, for example, the entrapment of nutrients and natural compounds such as probiotics which could aid and potentially protect their passage through the gastrointestinal tract (77). In the case of unsaturated fatty acids, microencapsulation can also help to improve their quality and shelf-life (78).

The unique process of microencapsulation may help to counteract traditional problems of strong odors/flavors (particularly those related to O3LC-PUFA delivery), reactions of other food matrix components which could reduce bioavailability and help to provide a form of targeted and controlled delivery and release (79). It is well recognized that food fortification with O3LC-PUFAs may be difficult due to their tendency to rapidly oxidize, variable bioavailability and poor water-solubility and encapsulation technologies may help to address some of these issues (80, 81). For example, research with flaxseed oil showed that the oxidative stability of encapsulated flaxseed oil was 13-fold higher than that in bulk oil form, demonstrating the stabilizing and protective effects of microencapsulation (82).

One mode of encapsulation involves integrating omega-3 oils into colloidal particles assembled from food-grade components such as emulsion droplets, liposomes, nanostructured lipid carriers, or microgels (80). Typical microencapsulation technologies can include approaches such as coacervation, extrusion, spray cooling and spray drying (78). Spray drying in particular is regarded as a flexible, simple and rapid microencapsulation method that is relatively easy to scale up (83). It is also regarded as a ‘clean technology’ as it does not utilize the use of organic solvents (83). Encapsulation has not been found to adversely affect the bioavailability of O3LC-PUFA compounds. In one double-blind trial 25 females were allocated to ingest 0.9 g n-3 PUFA/day in a capsule or microencapsulated fish-oil enriched foods (84). The microencapsulated fish-oil enriched foods were found to be as bioavailable as administration via a capsule thus regarded as an effective means of improving n-3 PUFA intakes to align with dietary recommendations (84). Foods such as ice cream and Indian yoghurt have also been found to administer microencapsulated flaxseed oils successfully (85, 86).

## Microalgae

It has previously been explained how seafood was traditionally exploited as a prime O3LC-PUFA source. However, other food derivates will be needed to meet expanding populations and growing global demands. Subsequently alternative sustainable non-animal derived sources of O3LC-PUFAs are being sought and algae is being recognized as one of these (87). It is now recognized that certain microalgae yields have significant levels of EPA and/or DHA (45). Subsequently, alternative microalgae sources could be a novel means to bridging gaps between supply and demand for EPA and DHA in relation to achieving human requirements (88).

Several clinical trials with micro-algae oil have led to significant increases in blood erythrocyte and plasma DHA (89–91). For example, Yang et al. (90) found that lactating women ingesting algal oil (200 mg/d) over 8 weeks significantly improved DHA levels in breast milk compared with a placebo capsule. Arteburn et al. (89) found that algal-oil capsules providing 600 mg/day DHA were bioequivalent in terms of providing similar levels of DHA to plasma and red blood cells when compared to cooked salmon. Earlier work by Geppert et al. (92) concluded that 0.94 g/d DHA over 8-weeks was well tolerated and could be a viable vegetarian O3LC-PUFA source. Sanders et al. (91) administered 1.5 g DHA + 0.6 g DPA to 79 healthy adults over 4 weeks finding that DHA and DPA erythrocyte phospholipid levels increased compared with the 4 g oil/d placebo.

## Discussion

Given rising sustainability and toxicological concerns in the aquaculture sector research into possible alternative sources of O3LC-PUFAs has become a priority, to help plug the gap between supply and demand (42, 88). Dietary intakes of O3LC-PUFAs largely remain to be insufficient in relation to the optimisation of health and cognitive outcomes (29, 30, 43). Historical evidence shows that fresh aquatic plants and seaweed were chewed such as red, green, and brown seaweeds and most likely consumed during Mesolithic and Neolithic time periods indicating that wild food resources were ingested before a switch to domesticated resources (93). These at the time were likely to have been an important source of nutrients, including omega-3 fatty acids.

Giving the scale of aging populations and surging healthcare costs attributed to poor brain health, reflection on insufficient omega-3 intakes and how to achieve these is needed (94). There is also growing evidence that DHA is an important neuroprotective agent potentially helping to enhance brain development, function, and maintenance (synaptic plasticity and cognition), particularly alongside exercise (95). Unfortunately, many supplements available do not enrich ‘brain’ DHA, although they may enrich most other body tissues (96). Intriguingly, research has found that DHA from triacylglycerol which is released as free DHA or monoacylglycerol during digestion and absorbed as triacylglycerol in chylomicrons is preferentially integrated into heart and adipose tissue but not the brain (96). In contrast, LPC lysophosphatidylcholine DHA has been found to enhance DHA uptake in the brain by up to 100% but does not affect adipose tissue (96).

Recently, findings from the REDUCE-IT trial remarkably found that EPA at a dose of 4 g/day in patients at high cardiovascular risk with hypertriglyceridemia had a 25% relative reduction in risk of cardiovascular-related events (97, 98). In terms of sources, omega-3 s from both dietary and supplementation sources may have positive health benefits, additionally, supplementation studies appear to demonstrate more consistent reductions in inflammatory markers including IL-6 and TNF-α amongst populations with mild cognitive impairment (99).

There has been some question regarding the bioavailability of O3LC-PUFAs from supplements which could have resulted in negative or neutral outcomes in some studies (38). Subsequently, this has led to the question as to whether novel models of delivery such as microencapsulation could improve omega-3 fatty acid bioavailability (38). Provisional science indicates that this could be a promising way forward, (76–79, 82). Ongoing research specifically focusing on O3LC-PUFAs is now needed.

Finally, it is important to consider that studies investigating novel methods to narrow omega-3 gaps should consider several factors. Firstly, studies measuring EPA and/or DHA status should recognize that plasma levels can change due to diurnal rhythm (100). Research with 21 adults aged 25 to 44 years found that rhythmicity was strongest for DHA which peaked in the evening at 17:43 (100). Circulating levels of EPA and DHA fatty acids fell during the night and reached the lowest point in the morning (100). The wider implications of this research indicate that O3LC-PUFA consumption in the evenings could have a wider functional significance (100). Secondly, other work has found that there appear to be O3LC-PUFAs “responders” and “non-responders” which could affect the results of human intervention studies (101). For example, it is speculated that certain epigenetic/genetic variants and differences in dietary and gut microbiota composition could be behind such variations (101). Finally, novel biomarkers such as brain derived neurotrophic factor (BDNF) could be more highly regarded than the omega-3 index when measuring omega-3 fatty acid brain enrichment as DHA is known to increase the synthesis of BDNF in the brain and there is bidirectional BDNF transport through the blood brain barrier (102).

Alongside this it should be considered that findings from studies investigating O3LC-PUFAs can vary due to short follow-up periods, small samples sizes, withdrawal rates, different types, timings, dosages, and forms of interventions. Baseline population characteristics also need to be carefully controlled and considered when making comparisons between studies. Greater consistency is needed when reporting such components to synthesize future evidence more effectively. Greater standardization between studies would greatly improve and strength any future conclusions.

## Conclusion

The present publication has identified modern-day challenges and shifting trends associated with inadequate O3LC-PUFA intakes and environmental challenges linked to meeting requirements for health via traditional means (oily fish consumption). The overarching conclusion is that novel models of EPA and DHA delivery could have a central role to play in helping to support future healthy and balanced diets whilst counteracting some of the sustainability and toxicological concerns that exist from present-day delivery methods.

## Author contributions

ED: Conceptualization, Visualization, Writing – original draft. CB: Writing – review & editing. GB: Writing – review & editing. AE: Writing – review & editing. PM: Writing – review & editing. WL: Writing – review & editing.
